# Bedaquiline, an FDA-approved antibiotic, inhibits mitochondrial function and potently blocks the proliferative expansion of stem-like cancer cells (CSCs)

**DOI:** 10.18632/aging.100983

**Published:** 2016-06-22

**Authors:** Marco Fiorillo, Rebecca Lamb, Herbert B. Tanowitz, Anna Rita Cappello, Ubaldo E. Martinez-Outschoorn, Federica Sotgia, Michael P. Lisanti

**Affiliations:** ^1^ The Breast Cancer Now Research Unit, Institute of Cancer Sciences, Cancer Research UK Manchester Institute, University of Manchester, Manchester, UK; ^2^ The Manchester Centre for Cellular Metabolism (MCCM), Institute of Cancer Sciences, Cancer Research UK Manchester Institute, University of Manchester, Manchester, UK; ^3^ The Department of Pharmacy, Health and Nutritional Sciences, The University of Calabria, Cosenza, Italy; ^4^ Departments of Pathology and Medicine, Albert Einstein College of Medicine, Bronx, NY 10461, USA; ^5^ The Sidney Kimmel Cancer Center, Philadelphia, PA 19107, USA; ^6^ School of Environment & Life Sciences, University of Salford, Salford, UK

**Keywords:** bedaquiline, mitochondria, tumor-initiating cells (TICs), cancer stem-like cells (CSCs), drug repurposing

## Abstract

Bedaquiline (a.k.a., Sirturo) is an anti-microbial agent, which is approved by the FDA for the treatment of multi-drug resistant pulmonary tuberculosis (TB). Bedaquiline is a first-in-class diaryl-quinoline compound, that mechanistically inhibits the bacterial ATP-synthase, and shows potent activity against both drug-sensitive and drug-resistant TB. Interestingly, eukaryotic mitochondria originally evolved from engulfed aerobic bacteria. Thus, we hypothesized that, in mammalian cells, bedaquiline might also target the mitochondrial ATP-synthase, leading to mitochondrial dysfunction and ATP depletion. Here, we show that bedaquiline has anti-cancer activity, directed against Cancer Stem-like Cells (CSCs). More specifically, we demonstrate that bedaquiline treatment of MCF7 breast cancer cells inhibits mitochondrial oxygen-consumption, as well as glycolysis, but induces oxidative stress. Importantly, bedaquiline significantly blocks the propagation and expansion of MCF7-derived CSCs, with an IC-50 of approx. 1-μM, as determined using the mammosphere assay. Similarly, bedaquiline also reduces both the CD44+/CD24low/− CSC and ALDH+ CSC populations, under anchorage-independent growth conditions. In striking contrast, bedaquiline significantly increases oxygen consumption in normal human fibroblasts, consistent with the fact that it is well-tolerated in patients treated for TB infections. As such, future pre-clinical studies and human clinical trials in cancer patients may be warranted. Interestingly, we also highlight that bedaquiline shares certain structural similarities with *trans*-piceatannol and *trans*-resveratrol, which are known natural flavonoid inhibitors of the mitochondrial ATP-synthase (complex V) and show anti-aging properties.

## INTRODUCTION

Residual treatment-resistant cancer cells are thought to be the drivers of poor clinical outcomes, in many cancer types [1]. These tumor-initiating cells (TICs) or cancer stem-like cells (CSCs) are resistant to conventional therapies, leading to recurrence, metastatic cell dissemination and drug-resistance [2-4]. CSCs are resistant to cellular stress, and are able to undergo anchorage-independent growth, allowing the formation of 3D multi-cellular tumor spheroids, that share properties with CSCs and progenitor cells [5, 6]. During these anchorage-independent conditions, most “bulk” epithelial cancer cells undergo cell death, termed “anoikis”. Thus, each 3D multi-cellular tumor-sphere is formed from the clonal expansion of a single CSC. As such, 3D tumor sphere formation enriches for a cell population with CSC-like properties [6]. In this context, 3D tumor spheres (i.e., mammospheres) prepared from breast cancer epithelial cell lines are a well-established model system.

We and others have recently shown that CSCs are critically dependent on mitochondrial function, for their successful propagation and clonal expansion [7-11]. In fact, during mammosphere formation, five subunits of the mitochondrial ATP synthase are significantly up-regulated (ATP5B, ATP5A1, ATP5F1, ATP5H and ATP5O); remarkably, ATP5B was dramatically over-expressed in MCF7-derived mammospheres, as compared with MCF7 monolayer cells [7]. Clinically, ATP5B protein levels are also elevated in the serum of patients with breast cancer and ATP5B is part of a 21-protein signature that predicts the development of distant metastasis [12].

During our continued search for FDA-approved drugs that may behave as mitochondrial inhibitors, we identified bedaquiline, an antibiotic that was originally developed to block the bacterial ATP-synthase of multidrug resistant pulmonary tuberculosis (TB) [13-15]. Because mitochondria originally evolved from bacteria, we speculated that bedaquiline might also target the mitochondrial ATP-synthase (Complex V), leading to ATP depletion.

Here, we propose that bedaquiline could be repurposed as a new anti-cancer drug, for the targeting in mitochondria within CSCs. More specifically, we show that bedaquiline treatment of MCF7 breast cancer cells inhibits oxygen-consumption and metabolically induces aerobic glycolysis (the Warburg effect), as well as oxidative stress. Importantly, bedaquiline blocks the propagation of MCF7-derived CSCs, with an IC-50 of 1 μM, as determined using the mammosphere assay. Bedaquiline also targets both CD44+/CD24low/− CSC and ALDH+ CSC populations, under anchorage-independent growth conditions.

## RESULTS

**Figure 1:**
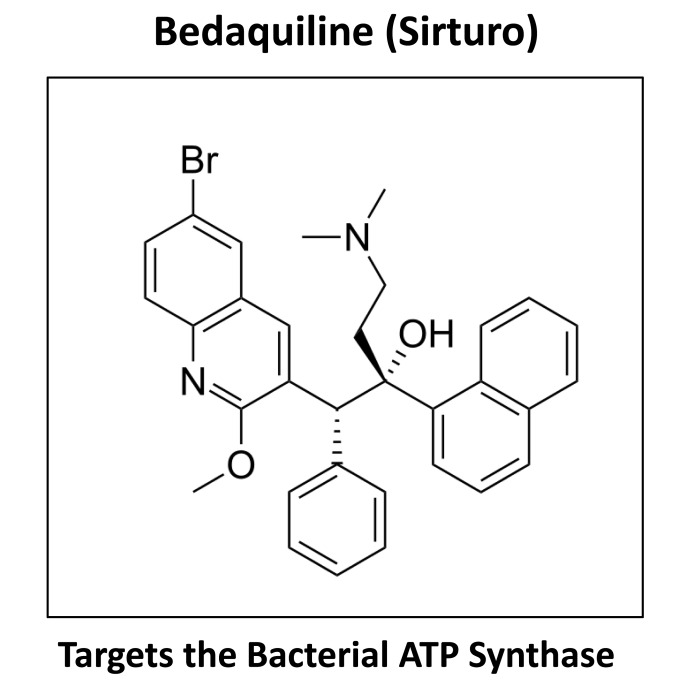
Bedaquiline: Structure and Activity Bedaquiline, also known as TMC207 and R207910, is a first-in-class diaryl-quinoline compound, that mechanistically inhibits the bacterial ATP-synthase.

Here, we aimed to assess the mitochondrial inhibitory effects of bedaquiline (also known as TMC207 and R207910). In addition, we tested the hypothesis that bedaquiline could be used to inhibit the propagation of CSCs. The structure of bedaquiline is shown in Figure 1.

### Metabolic profiling of MCF7 breast cancer cells treated with bedaquiline

First, we investigated the metabolic effects of bedaquiline on MCF7 breast cancer cells grown as monolayers. Extracellular acidification rates (ECAR) and real-time oxygen consumption rates (OCR) for cells treated with bedaquiline were assessed using the Seahorse Extracellular Flux (XFe-96) analyzer. More specifically, OCR is a surrogate marker for OXPHOS activity, while ECAR is a measure of glycolysis.

**Figure 2:**
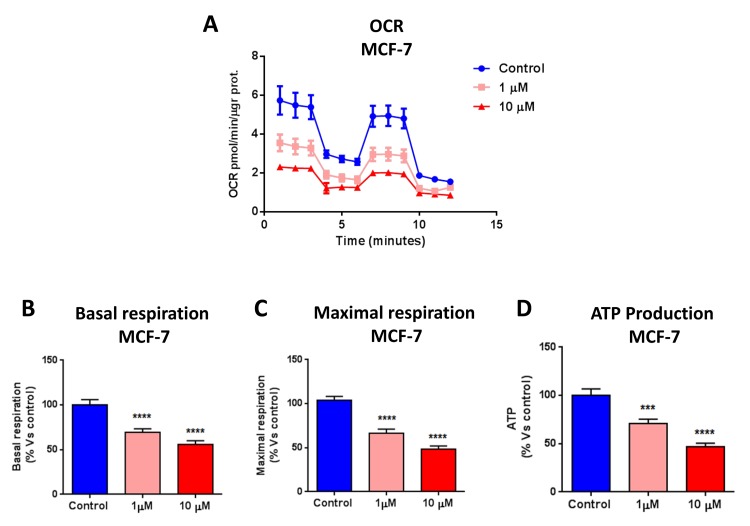
Bedaquiline greatly diminishes oxygen consumption in MCF7 breast cancer cells The Seahorse XF96 analyzer was employed to determine the mitochondrial function of MCF7 cells treated with bedaquiline (1μM and 10 μM) for 48 hours. (**A**) A line graph of 3 independent experiments is shown. (**B, C, D**) Respiration (basal and maximal), as well as ATP levels, were significantly decreased. *** p<0.0001, **** p<0.00001.

**Figure 3:**
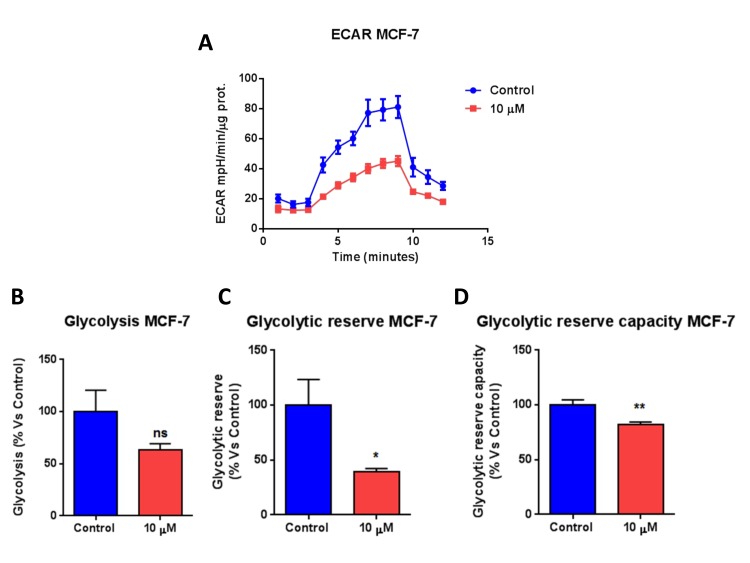

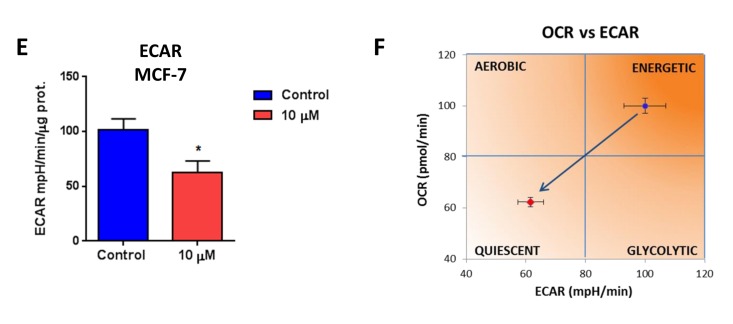
Bedaquiline greatly diminishes glycolysis in MCF7 breast cancer cells The Seahorse XF96 analyzer was employed to determine the status of glycolysis in MCF7 cells treated with 10μM bedaquiline for 48 hours. (**A**) A line graph of 3 independent experiments is shown. (**B, C, D, E**) Note the reductions in glycolytic reserve, glycolytic reserve capacity and ECAR. (**F**) The plot of OCR versus ECAR shows that bedaquiline shifts MCF7 cells from an energetic to a quiescent state. * p<0.01, ** p<0.001, ns not significant.

Figure 2 shows that bedaquiline dose dependently inhibits oxygen consumption in MCF7 cells, reducing respiration, both maximal and basal, as well as ATP levels. In addition, bedaquiline inhibited glycolysis in MCF7 cells (Figure 3, A-E). Determination of the Cell Energy Profile indicated that bedaquiline shifts MCF7 cells from an energetic to a quiescent state (Figure 3F).

**Figure 4:**
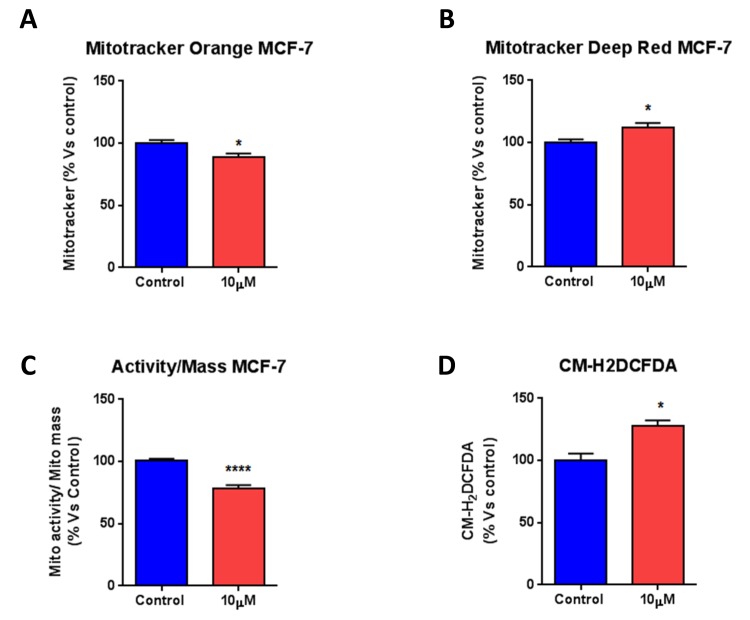
Bedaquiline reduces mitochondrial membrane potential, with a significant rise in ROS levels FACS analysis was carried out on MCF7 cells treated with 10μM bedaquiline for 48 hours. (**A, B**) Bedaquiline reduces mitochondrial membrane potential (MitoTracker Orange), with a likely compensatory increase in mitochondrial mass (MitoTracker Deep-Red). (**C**) The ratio of mitochondrial membrane potential to mass indicates that bedaquiline reduces the membrane potential per mitochondria. (**D**) Cells were stained with the CM-H_2_DCFDA probe and analyzed by FACS to determine ROS levels. Note that ROS levels are increased by bedaquiline treatment. * p<0.01, **** p<0.00001.

Next, we performed FACS analysis on MCF7 cells stained with MitoTracker probes to follow mitochondrial mass and membrane potential. Figure 4A shows that bedaquiline reduces MitoTracker Orange staining, indicative of decreased mitochondrial membrane potential. Conversely, Figure 4B shows that bedaquiline increases MitoTracker Deep-Red staining, reflecting increased mitochondrial mass. Importantly, assessing the ratio of mitochondrial membrane potential to mass revealed that bedaquiline significantly reduces membrane potential per mitochondria (Figure 4C). Finally, Reactive Oxygen Species (ROS) levels were increased, as assessed by FACS analysis using the CM-H_2_DCFDA probe (Figure 4D). In summary, these data provide direct evidence that bedaquiline functionally targets mitochondria by inhibiting oxygen consumption in human breast cancer cells, driving an increase in oxidative stress and glycolytic function.

### Metabolic profiling of normal human fibroblasts (hTERT-BJ1) treated with bedaquiline

**Figure 5:**
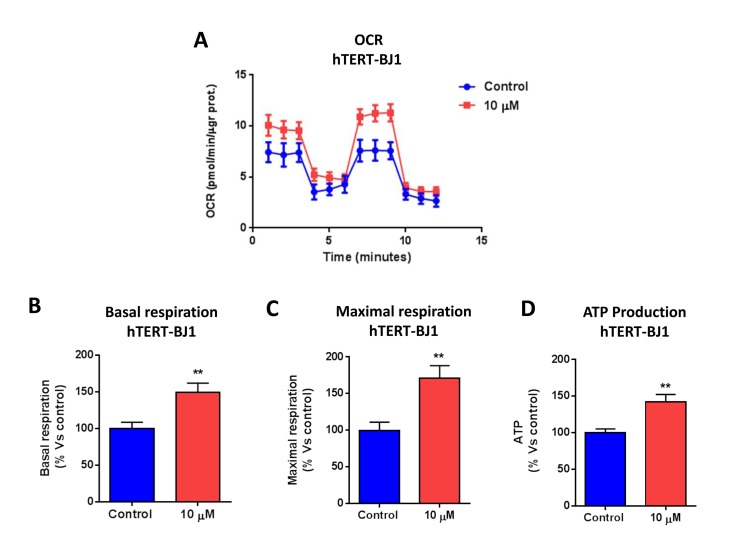
Bedaquiline augments oxygen consumption in normal human fibroblasts The Seahorse XF96 analyzer was employed to determine the mitochondrial function of MCF7 cells treated with 10 μM bedaquiline for 48 hours. (**A**) The line graph of 3 independent experiments is shown. (**B, C, D**) Basal respiration, maximal respiration, and ATP levels were all increased by bedaquiline treatment of normal fibroblasts. ** p<0.001.

**Figure 6:**
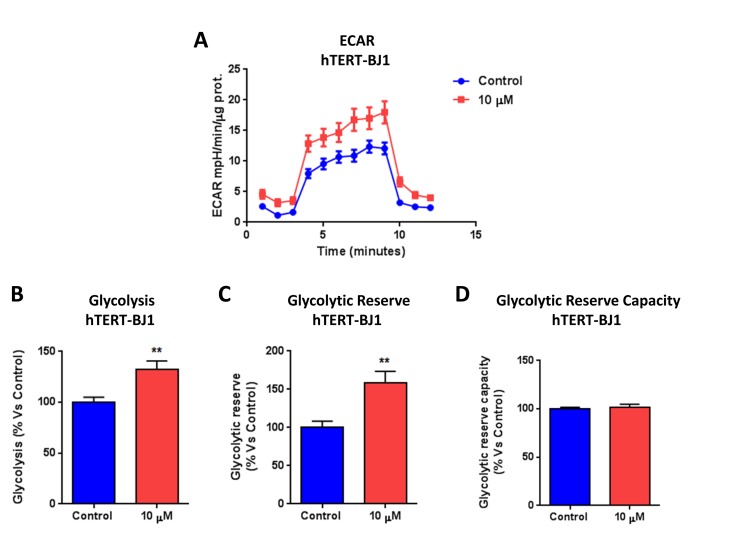

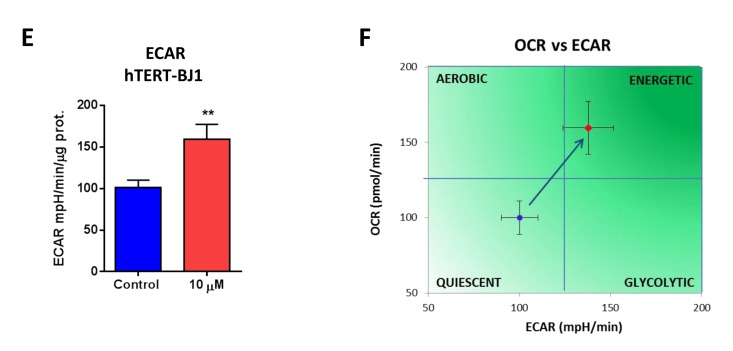
Bedaquiline augments glycolysis in normal human fibroblasts The Seahorse XF96 analyzer was employed to determine the status of glycolysis in normal human fibroblasts treated with 10μM bedaquiline for 48 hours. (**A**) The line graph of 3 independent experiments is shown. (**B, C, D, E**) Glycolysis, glycolytic reserve, and ECAR were elevated. **F**. The plot of OCR versus ECAR shows that bedaquiline shifts the metabolic state of normal fibroblasts from a quiescent state to an energetic state. ** p<0.001.

To assess whether bedaquiline also behaves as a mitochondrial inhibitor in normal human fibroblasts, we examined its metabolic effects on hTERT-BJ1 cells, after 48 hours treatment. Interestingly, Figure 5 demonstrates that bedaquiline augments oxygen consumption and ATP levels in normal human fibroblasts, as determined by using the Seahorse XF-e96 analyzer. In addition, bedaquiline also boosts glycolysis in normal human fibroblasts, as seen in Figure 6. Remarkably, the Cell Energy Profile plot (OCR versus ECAR) shows that bedaquiline shifts normal human fibroblasts from a quiescent to an energetic state (Figure 6F), improving their overall metabolic profile. Thus, our results firmly establish that bedaquiline specifically inhibits mitochondrial respiration in cancer cells, while enhancing mito-chondrial function in normal cells.

### Bedaquiline significantly inhibits CSC propagation and survival

Since bedaquiline behaved as a specific inhibitor of mitochondrial respiration in cancer cells, we next examined its effects on the behavior of the CSC population.

**Figure 7:**
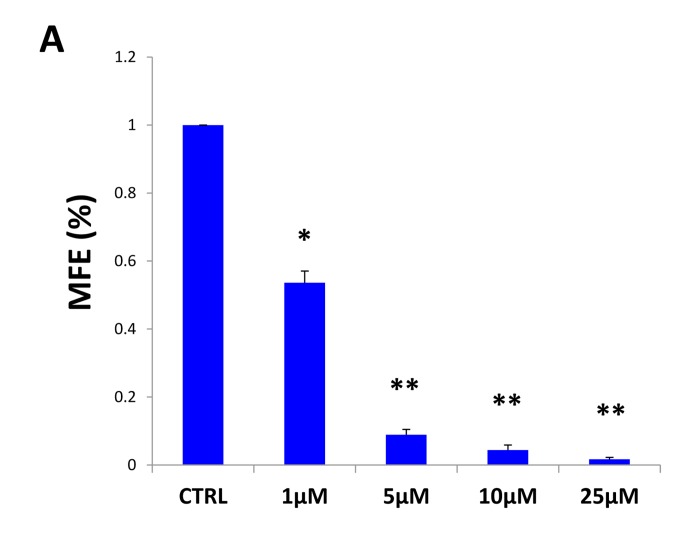

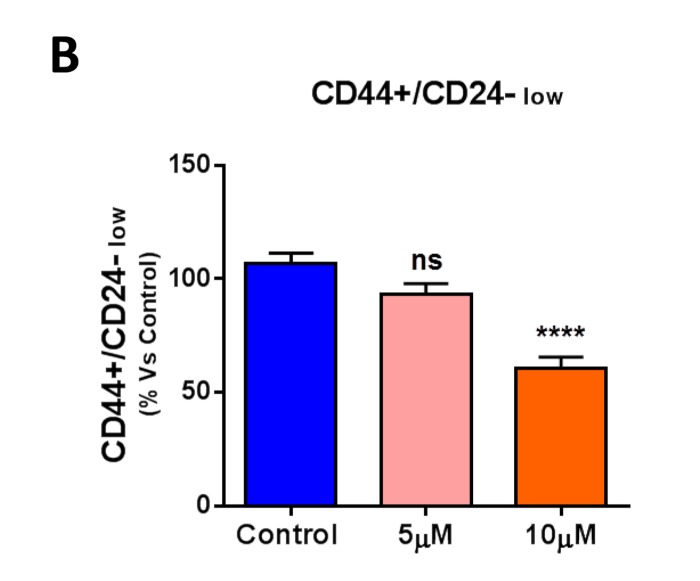

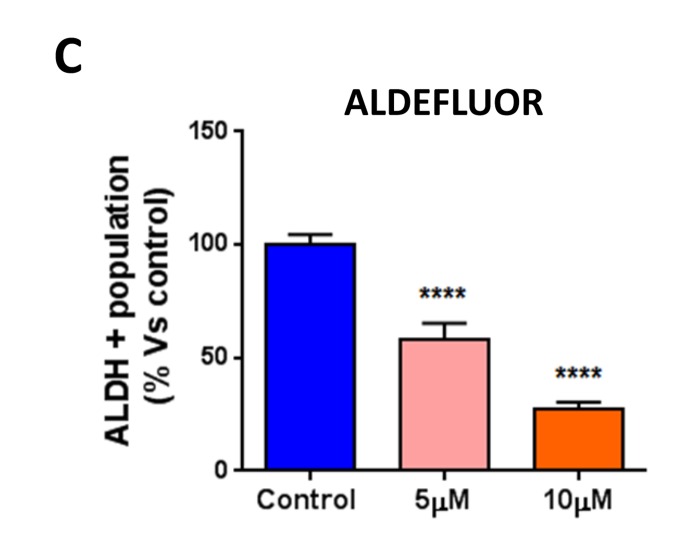

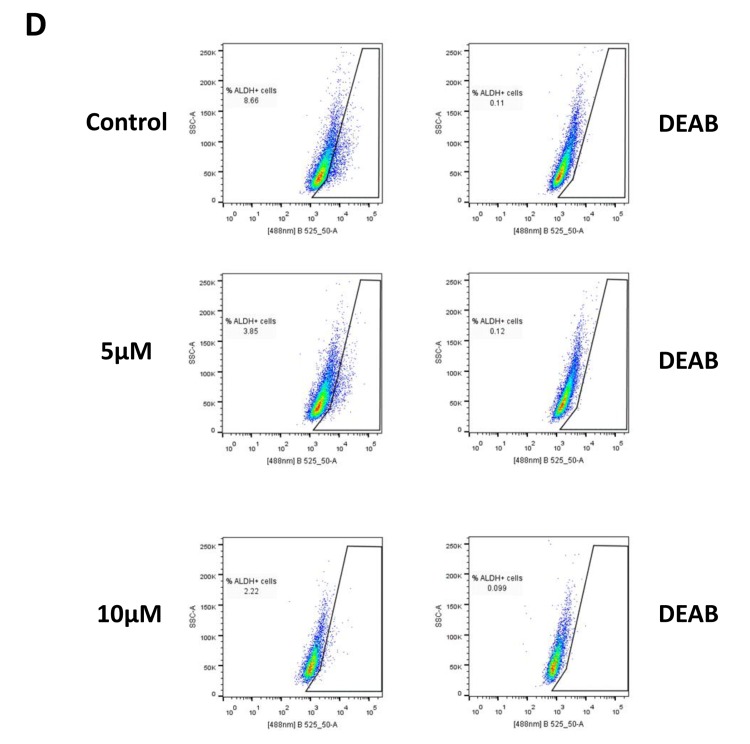
Bedaquiline halts the propagation of the CSC population (**A**) MCF7 cells were grown as mammospheres, and treated with bedaquiline at increasing concentrations or vehicle alone. Note that bedaquiline inhibits mammosphere formation efficiency (MFE), in a dose-dependent fashion, with an IC-50 of ~1 μM. * p<0.05, ** p< 0.01, evaluated by Student's t test. (**B**) MCF7 cell monolayers were treated with bedaquiline (5μM and 10 μM) for 48 hours and then re-plated on low-attachment plates in the absence of bedaquiline for 12 hours. The expression of CD44 and CD24 (CSC markers) was analyzed by FACS. Note that the CD44(+)high/CD24(−)low cell population, which is believed to be the CSC population, is reduced by bedaquiline. (**C, D**) Similar results were also obtained using ALDEFLOUR as a marker of CSCs. ALDEFLUOR was measured by FACS analysis. DEAB (diethylaminobenzal-dehyde), a specific ALDH inhibitor, was used as a negative control for each sample. **** p<0.00001.

More specifically, MCF7 cells were grown as mammospheres and treated with bedaquiline at increasing concentrations. Vehicle alone controls were processed in parallel. Interestingly, Figure 7A shows that bedaquiline halts mammosphere formation in a dose-dependent fashion (IC-50 ~1 μM). In addition, expression levels of CSC markers (CD24 and CD44) were determined. Under these anchorage-independent conditions, bedaquiline diminished the CD44+/CD24− ^low^ cancer cell population, which are believed to be the tumor-initiating cells (Figure 7B).

Finally, we evaluated the levels of ALDEFLUOR, an independent marker of CSCs. ALDEFLUOR was measured by FACS analysis. Figure 7C shows that bedaquiline significantly decreases the ALDEFLUOR-positive cell population. As a negative control, we employed DEAB (diethylaminobenzaldehyde), a specific ALDH inhibitor (Figure 7D). Thus, these results indicate that bedaquiline pre-treatment reduces the CSC population, as assessed by three independent parameters, namely mammosphere formation, CD44/CD24 staining and ALDEFLUOR activity.

### Bedaquiline does not affect the viability of the total cancer cell population, or normal human fibroblasts

**Figure 8:**
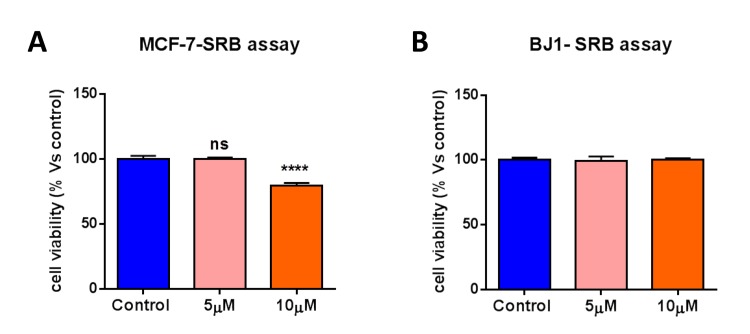
Bedaquiline has little or no effect on the viability of MCF7 cancer cells and normal human fibroblasts Bedaquiline (5μM and 10 μM) treatment of MCF7 cells and hTERT-BJ1 normal human fibroblasts was carried out for 5 days. The SRB assay was performed as a measure of cell viability. (**A, B**) Bedaquiline has little or no inhibitory effects on the viability of MCF7 cells and human fibroblasts. **** p<0.00001, ns not significant.

We next determined if bedaquiline targets only the CSC-population, or if bedaquiline generally reduces the survival of the ‘bulk’ MCF7 cancer cell population and/or normal human fibroblasts (hTERT-BJ1). Figure 8A shows that bedaquiline treatment had little or no effect on MCF7 cell viability, even at a concentration of 10 μM. Similarly, bedaquiline treatment had no effect on the viability of normal human fibroblasts (Figure 8B). In summary, our results indicate that bedaquiline preferentially inhibits the CSC population (compare Figures 7 and 8), relative to the ‘bulk’ cancer cells and fibroblasts.

### Bedaquiline does not induce a stress response in normal human fibroblasts

**Figure 9:**
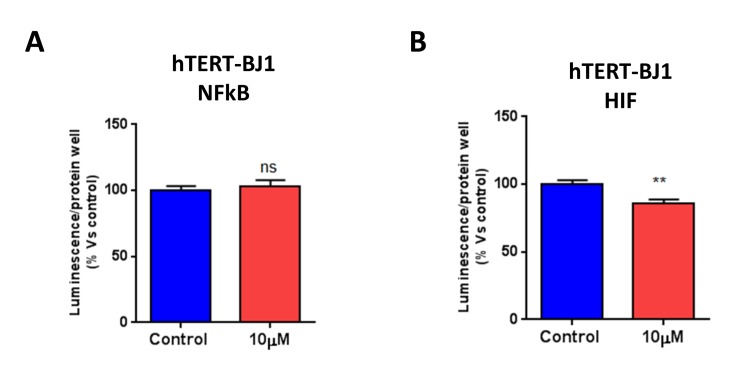
Bedaquiline does not stimulate a stress-response in normal fibroblasts (**A**, **B**) Luc-reporter fibroblasts were employed to assess HIF and NFκB activation. Cells treated with 10 μM bedaquiline or DMSO control for 48 hours were analyzed by luciferase assays. Luminescence was normalized by SRB (total proteins). Bedaquiline does not activate NFκB pathway (**A**) in normal fibroblasts, while slightly reducing HIF activation (**B**) ** p<0.001, ns not significant.

The increased ROS levels observed in MCF7 cells (Figure 4D) after bedaquiline treatment may also induce oxidative stress, activating key signaling pathways, such as HIF and NFκB transcription factors. Activation of these pathways was assessed by employing luciferase reporter assays. Figure 9 (A and B) shows that bedaquiline treatment did not affect the activation status of the NFκB pathway in normal fibroblasts, and it only slightly decreased HIF signaling, under these conditions. These results indicate that bedaquiline does not induce a significant stress response, in normal human fibroblasts.

### Validation of the prognostic value of ATP5B in human breast cancer patients, in both ER(+) and ER(−) sub-types

Clinically, ATP5B protein levels are elevated in the serum of patients with breast cancer and ATP5B is part of a 21-protein signature that predicts the development of distant metastasis [12]. Here, we further validated the prognostic value of ATP5B. More specifically, we assessed whether the levels of ATP5B mRNA show any prognostic value in human breast cancer patient cohorts, with long-term follow-up (approaching 15-20 years). Results are summarized in Table 1. High mRNA expression levels of ATP5B were clinically associated with significantly reduced overall survival. Similar results were obtained in both ER(+) and ER(−) patients, as well as in ER(+) sub-types (Table 1). For specific Kaplan-Meier (K-M) curves, please see Supplemental Figure S1. Thus, we independently confirmed the prognostic value of ATP5B, a key subunit of mito-chondrial complex V. As such, elevated levels of ATP5B could possibly be used to identify breast cancer patients that might benefit from treatment with bedaquiline.

**Table 1:** High ATP5B mRNA levels are associated with decreased overall survival, in both ER(+) and ER(−) breast cancer patients

Cancer Subtype	HR/OS	Log-Rank	N
All Subtypes	1.64	8.3e-05	1,103
			
ER(+)	1.77	7.4e-05	819
ER(−)/Basal	2.41	0.019	198
			
ER(+)/Luminal A	1.63	0.039	500
ER(+)/Luminal B	1.98	0.001	319

ER, estrogen receptor; HR, hazard ratio; OS, overall survival; N, the number of patients in a given subtype or group. Overall survival data spans from 15-20 years post-diagnosis.

## DISCUSSION

Bedaquiline is an FDA-approved anti-microbial drug that was developed by Janssen Pharmaceuticals for the treatment of multi-drug resistant pulmonary tuber-culosis (TB) [14]. It was first approved by the FDA at the end of 2012. Bedaquiline mechanistically inhibits the bacterial ATP synthase [13]. Thus, we hypothesized that, in cancer cells, bedaquiline might also target the mitochondrial ATP-synthase, leading to mitochondrial dysfunction and ATP depletion. Here, we explored the possibility that bedaquiline could be repurposed for targeting mitochondrial complex V in breast cancer cells. To test this hypothesis, we determined its metabolic effects on MCF7 cells, using the Seahorse XF-e96, to measure mitochondrial function and glycolysis. Our results demonstrate that bedaquiline dramatically inhibits oxygen consumption and ATP production in the low micromolar range. In addition, bedaquiline treatment also reduced aerobic glycolysis (a.k.a., the Warburg effect). In this context, bedaquiline reduced mitochondrial membrane potential, but increased mitochondrial mass and overall ROS production. Most importantly, bedaquiline potently inhibited the propagation of CSCs, with an IC-50 of 1 μM, as measured using the mammosphere assay. Remarkably, bedaquiline treatment increased mitochondrial respiratory function, ATP production, and glycolysis in normal human fibroblasts. Thus, these results provide clear evidence that bedaquiline specifically inhibits mitochondrial respiration in MCF7 cancer cells, but not in normal human fibroblasts. As such, our new findings suggest that bedaquiline could be used to target the mitochondrial F0F1-ATPase/ATP synthase in CSCs.

Consistent with our current findings, we have previously shown that oligomycin, an established inhibitor of the mitochondrial ATP-synthase (complex V), effectively targets MCF7-derived CSCs with an IC-50 of 100 nM [7]. However, oligomycin (another antibiotic) in not FDA-approved, and is considered to be both a bacterial and mitochondrial “poison”, that is too toxic for safe use in humans or animals.

Interestingly, only one previous study showed that TMC207 (now known as bedaquiline) had little or no effect on ATP production in a human ovarian cancer cell line, namely OVCAR3 cells, with an IC-50 > 200 μM [16]. However, no other cancer cell lines were studied. Similarly to our results, bedaquiline had minimal inhibitory activity on oxygen consumption in mouse liver and bovine heart mitochondria. The authors thus suggested that the inhibitory effects of bedaquiline were restricted to mycobacterium. Importantly, the effects of bedaquiline on cancer stem-like cells were not evaluated [16]. It has been suggested that bedaquiline binds to the subunit C of the mycobacterial ATP synthase, based on computational modeling [17]. This corresponds to the three human genes, namely ATP5G1, ATP5G2, ATP5G3, which differ only in their mitochondrial import sequence, but not in the sequence of the mature protein.

Interestingly, several natural products, especially polyphenolic phytochemicals [18], also act as inhibitors of the F0F1-ATPase/ATP synthase (Complex V), that is present within the inner membrane of eukaryotic mitochondria. These include the following compounds, listed in rank order of their potency: piceatannol > resveratrol > epigallocatechin-gallate >epicatechin-gallate/curcumin > genistein/bio-chanin A/quercetin/kaempferol/morin> phloretin/apigenin/daidzein.

**Figure 10:**
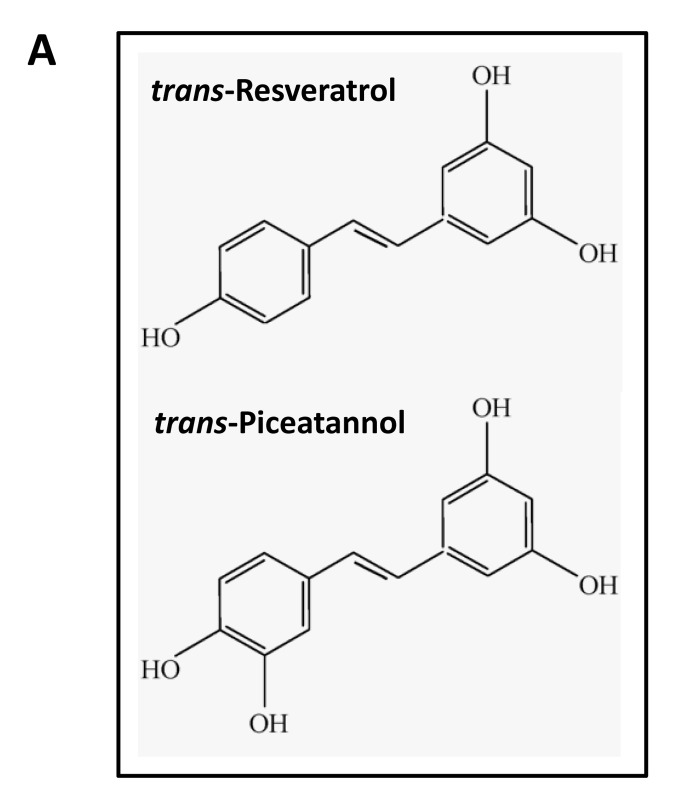

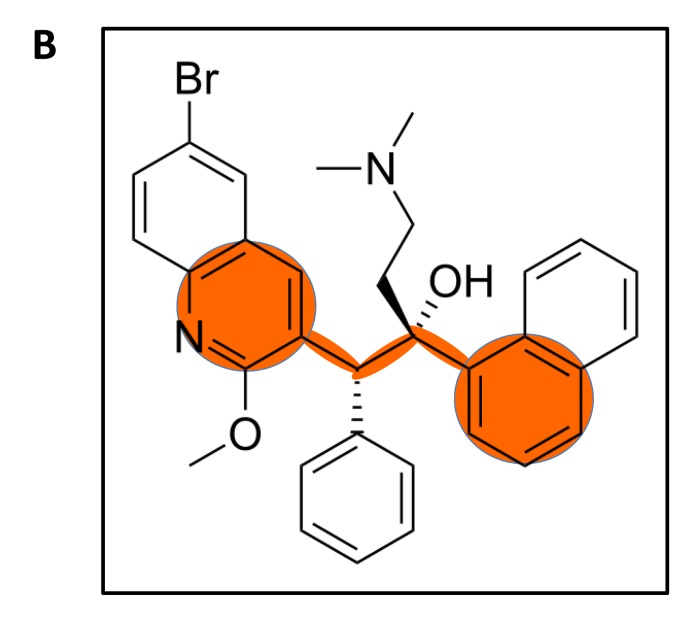
Structure of trans-Piceatannol and trans-Resveratrol (**A**) Interestingly, these polyphenolic phytochemicals act as inhibitors of the F0F1-ATPase/ATP synthase (Complex V), that is present within the inner membrane of eukaryotic mitochondria. These compounds have been suggested to act as either anti-cancer agents or as compounds that have anti-aging properties. (**B)** Structural similarities with the bedaquiline molecule are highlighted in orange. Note that the structural backbone of trans-piceatannol and trans-resveratrol is actually embedded within bedaquiline.

The structures of some of these compounds are highlighted in Figure 10A. Many of these flavonoid compounds have been suggested to act as either anti-cancer agents or as compounds that have anti-aging properties, consistent with their effects of reducing or “fine-tuning” mitochondrial function [18, 19].

Interestingly, Figure 10B shows that the structural backbone of trans-piceatannol and trans-resveratrol is actually embedded within bedaquiline. These common structural similarities within the bedaquiline molecule are specifically highlighted in orange. In fact, *trans-*piceatannol and *trans-*resveratrol also functionally demonstrate anti-bacterial properties, behaving as antibiotics [19].

**Figure 11:**
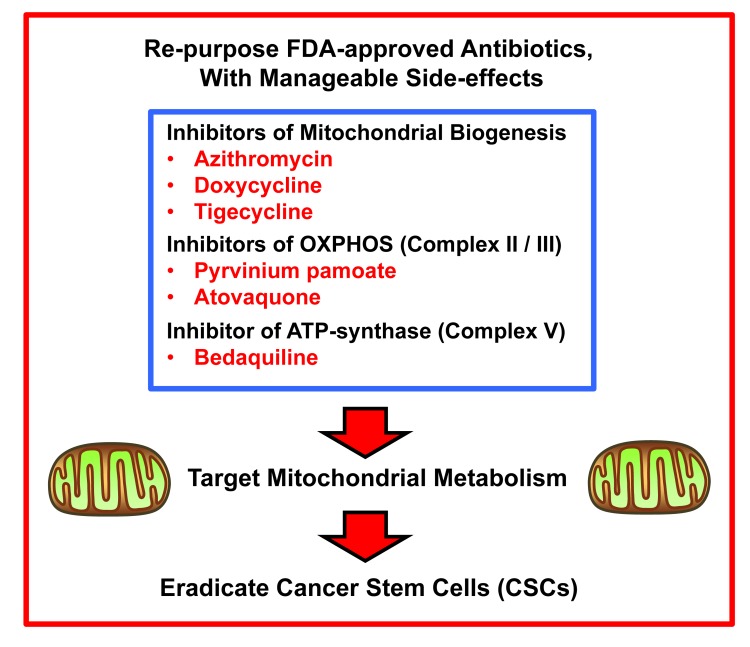
Summary: Overall strategy for repurposing FDA-approved antibiotics as anti-cancer agents for targeting mitochondria in CSCs Here, show that bedaquiline, an FDA-approved antibiotic, effectively prevents the propagation of CSCs, by targeting oxidative mitochondrial metabolism. Thus, the repurposing of antibiotics, to target mitochondria in CSCs is a novel strategy for accelerating clinical trials, as these FDA-approved drugs have already passed Phase I clinical trials, and could be used in Phase II clinical trials, such as Window-of-Opportunity studies. So far, we have identified six different classes of FDA-approved antibiotics that fit this profile.

Our overall approach to drug repurposing is briefly summarized in Figure 11. Thus far, we have identified six different classes of FDA-approved antibiotics that could be used to therapeutically target mitochondria in CSCs. As of yet, we do not know the underlying mechanism(s) of selectivity that allows the inhibitory effects of bedaquiline to be seen preferentially at the level of the CSC, but not in normal fibroblasts and to a lesser extend in mature cancer cells. One possibility is that the mitochondrial ATPase (Complex V) in cancer cells and CSCs undergoes a conformational change, so that its 3-D structure more closely resembles the ancient bacterial ATPase. This hypothesis deserves further investigation.

## MATERIALS AND METHODS

### Materials

MCF7 breast cancer cell lines were purchased from the ATCC. Human immortalized fibroblasts (hTERT-BJ1) were originally purchased from Clontech, Inc. Cells were cultured in Dulbecco's modified Eagle's medium (DMEM), supplemented with 10% FBS (fetal bovine serum), 2 mM GlutaMAX, and 1% Pen-Strep in a 37°C humidified atmosphere containing 5% CO_2_, unless other-wise noted. Gibco-brand cell culture media (DMEM/F12) was purchased from Life Technologies. Bedaquiline was purchased from AdooQ Bioscience.

### Seahorse XFe-96 metabolic flux analysis

Extracellular acidification rates (ECAR) and real-time oxygen consumption rates (OCR) for cells treated with bedaquiline were assessed using the Seahorse Extracellular Flux (XFe-96) analyzer (Seahorse Bioscience, MA, USA) [20]. 10,000 cells per well were seeded into XFe-96 well cell culture plates, and incubated overnight to allow attachment. Cells were then treated with bedaquiline (1μM or 10 μM) for 48 hours. Vehicle alone (DMSO) control cells were processed in parallel. After 48 hours of incubation, cells were washed in pre-warmed XF assay media (or for OCR measurement, XF assay media supplemented with 10mM glucose, 1mM Pyruvate, 2mM L-glutamine and adjusted at 7.4 pH). Cells were then maintained in 175 μL/well of XF assay media at 37°C, in a non-CO_2_ incubator for 1 hour. During the incubation time, we loaded 25 μL of 80mM glucose, 9μM oligomycin, and 1M 2-deoxyglucose (for ECAR measurement) or 10μM oligomycin, 9μM FCCP, 10μM rotenone, 10μM antimycin A (for OCR measurement), in XF assay media into the injection ports in the XFe-96 sensor cartridge. Measurements were normalized by protein content (Bradford assay). Data set was analyzed by XFe-96 software and GraphPad Prism software, using one-way ANOVA and Student's t-test calculations. All experiments were performed in quintuplicate, three times independently. Energy plots, shown in Figures 3F and 6F, were generated by following the manufacturer's guidelines and instructions (Seahorse, Inc.).

### Mitochondrial staining

Mitochondrial activity was assessed with MitoTracker Orange (#M7510, Invitrogen), whose accumulation in mitochondria is dependent upon membrane potential. Mitochondrial mass was determined using MitoTracker Deep-Red (#M22426, Invitrogen), localizing to mitochondria regardless of mitochondrial membrane potential. MCF7 cells were treated with 10 μM bedaquiline for 48 hours. Vehicle alone (DMSO) control cells were processed in parallel. After 48 hours, cells were incubated with pre-warmed MitoTracker staining solution (diluted in PBS/CM to a final concentration of 10 nM) for 30-60 minutes at 37°C. All subsequent steps were performed in the dark. Cells were washed in PBS, harvested, and re-suspended in 300 μL of PBS/CM. Cells were then analyzed by flow cytometry. Data analysis was performed using FlowJo software.

### ROS staining

Reactive oxygen species (ROS) production was measured using CM-H_2_DCFDA (C6827, Invitrogen), a cell-permeable probe that is non-fluorescent until oxidation within the cell. MCF7 cells were treated with 10μM bedaquiline for 48 hours. Vehicle alone (DMSO) control cells were processed in parallel. After 48 hours, cells were washed with PBS, and incubated with CM-H_2_DCFDA (diluted in PBS/CM to a final concentration of 1 μM) for 20 minutes at 37°C. All subsequent steps were performed in the dark. Cells were rinsed, harvested, and re-suspended in PBS/CM. Cells were then analyzed by flow cytometry using the Fortessa (BD Bioscience). ROS levels were estimated by using the mean fluorescent intensity of the viable cell population. The results were analyzed using FlowJo software (Tree star Inc.).

### Mammosphere culture

A single cell suspension was prepared using enzymatic (1x Trypsin-EDTA, Sigma Aldrich, #T3924), and manual disaggregation (25 gauge needle) to create a single cell suspension [6]. Cells were plated at a density of 500 cells/cm^2^ in mammosphere medium (DMEM-F12/B27/20ng/ml EGF/PenStrep) in non-adherent conditions, in culture dishes coated with (2-hydroxyethylmethacrylate) (poly-HEMA, Sigma, #P3932). Then, cells were treated with increasing concentrations of bedaquiline (range 1 μM to 25 μM). Vehicle alone (DMSO) control cells were processed in parallel. Cells were grown for 5 days and maintained in a humidified incubator at 37°C. After 5 days for culture, spheres >50 μm were counted using an eye piece graticule, and the percentage of cells plated which formed spheres was calculated and is referred to as percentage mammosphere formation, and was normalized to one (1 = 100 %MFE). Similar results were also obtained when cells were seeded at a density of 200 cells/cm^2^.

### CD44/CD24 analysis

1 × 10^5^ MCF7 cells were treated with bedaquiline (5 μM and 10 μM) for 48 hours in 6-well plates, grown as a monolayer. Then, cells were trypsinized and seeded in low-attachment plates in mammosphere media. After 12 hours, MCF7 cells were spun down and incubated with CD24 (IOTest CD24-PE, Beckman Coulter) and CD44 (APC mouse Anti-Human CD44, BD Pharmingen cat.559942) antibodies for 15 minutes on ice. Cells were rinsed twice and incubated with LIVE/DEAD dye (Fixable Dead Violet reactive dye; Invitrogen) for 10 minutes. Samples were then analyzed by FACS (Fortessa, BD Bioscence). Only the live population, as identified by the LIVE/DEAD dye staining, was analyzed for CD24/CD44 expression. Data were analyzed using FlowJo software.

### SRB assay

Cell viability was assessed by sulphorhodamine (SRB) assay, based on the measurement of cellular protein content. After treatment with bedaquiline (5μM and 10 μM) for 5 days in 96 well plates, cells were fixed with 10% trichloroacetic acid (TCA) for 1 hour in cold room, and dried overnight at room temperature. Then, cells were incubated with SRB for 15 min, washed twice with 1% acetic acid, and air dried for at least 1 hour. Finally, the protein-bound dye was dissolved in 10 mM Tris pH 8.8 solution and read using a plate reader at 540 nm.

### Evaluation of HIF and NFκB signaling pathways

The Cignal Lenti reporter assay (Luc) system (Qiagen) was chosen for monitoring HIF- and NFκB-Luc pathway activity in fibroblasts, as we previously described [21] [22]. Luciferase assay (E1501, Promega) was performed in luciferase reporter hTERT-BJ1 cells treated with bedaquiline. Briefly, 1 × 10^4^ hTERT-BJ1 reporter cells were seeded in black-walled 96-well plates and then were treated with 10 μM bedaquiline for 48 hours. Vehicle alone (DMSO) control cells were run in parallel. Three replicates were used for each condition. After 48 hours, luciferase assay was performed according to the manufacturer's instructions. Light signal was acquired for 2 minutes in photons/second in the Xenogen VivoVision IVIS Lumina (Caliper Life Sciences), and the results were analyzed using the Living Image 3.2 sofware (Caliper Life Sciences). Luminescence was normalized using SRB (total proteins), as a measure of cell viability.

### Statistical analysis

Data is represented as the mean ± standard error of the mean (SEM), taken over ≥ 3 independent experiments, with ≥ 3 technical replicates per experiment, unless otherwise stated. Statistical significance was measured using the analysis of variance (ANOVA) test or student t-test. P ≤ 0.05 was considered significant and all statistical tests were two-sided.

### Validating the prognostic value of ATP5B

We used an open-access online survival analysis tool to interrogate publically available microarray data from up to 1,103 breast cancer patients [23]. Hazard-ratios were calculated, at the best auto-selected cut-off, and p-values were calculated using the logrank test and plotted in R. K-M curves were generated online using the K-M-plotter (as high-resolution TIFF files), using univariate analysis: http://kmplot.com/analysis/index.php?p=service&cancer = breast.

## SUPPLEMENTARY MATERIAL FIGURE


