# Supplementary material for: Bedaquiline, an FDA-approved antibiotic, inhibits mitochondrial function and potently blocks the proliferative expansion of stem-like cancer cells (CSCs)
Source: Aging (Albany NY). 2016 Jun 22;8(8):1593–606. doi: 10.18632/aging.100983 (PMC5032685; doi:10.18632/aging.100983)
Supplement: Supplementary file 1 [file aging-08-1593-s001.pdf]

SUPPLEMENTARY MATERIAL

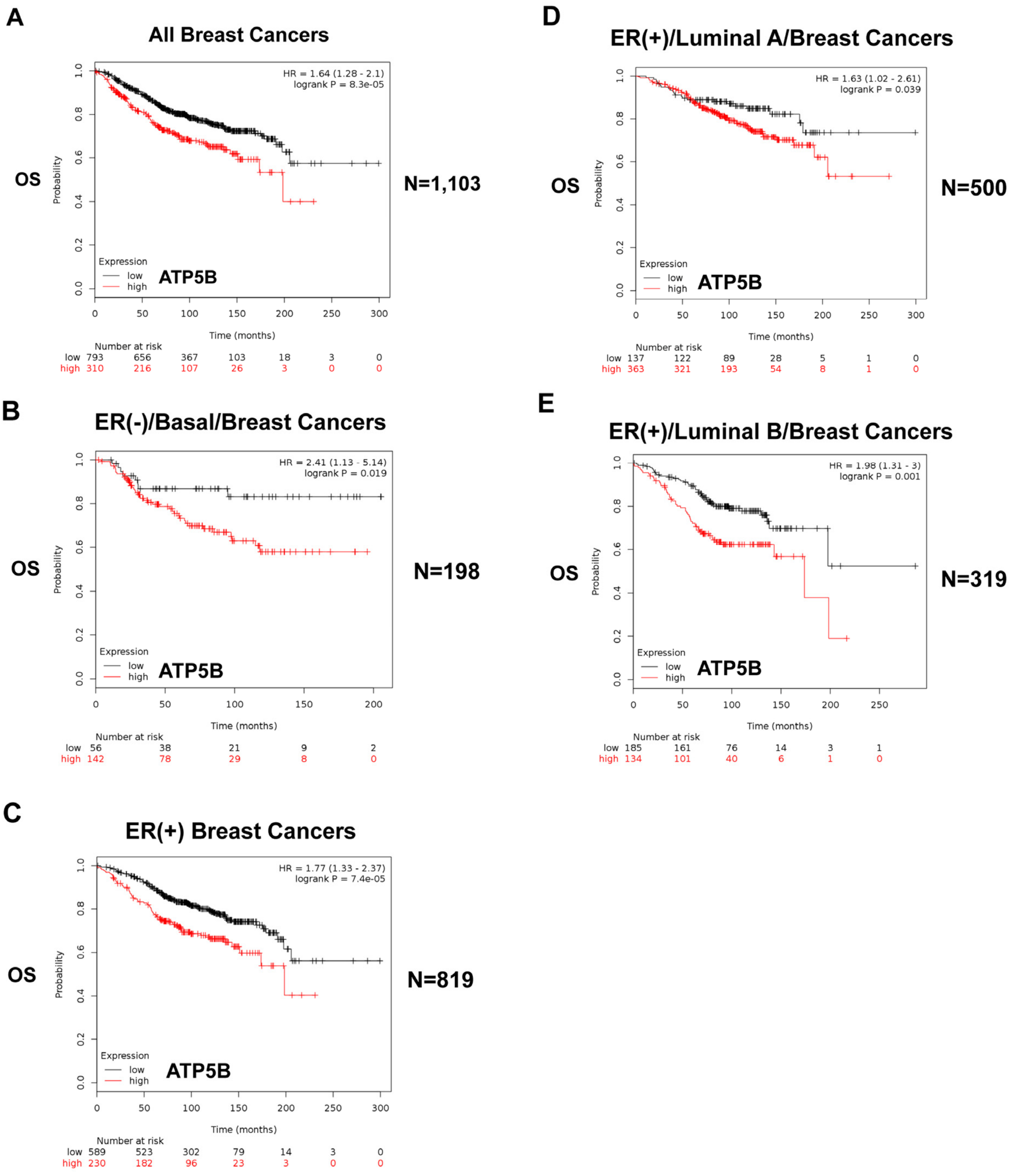

Supplementary Figure S1. K-M plots of OS (Overall Survival) are shown for ATP5B expression in different sub-groups of breast cancer patients (Panels **A**-**E**).
